# Microwave Assisted Reduction of Pt-Catalyst by *N*-Phenyl-*p*-Phenylenediamine for Proton Exchange Membrane Fuel Cells

**DOI:** 10.3390/polym9030104

**Published:** 2017-03-15

**Authors:** Ming-Jer Tsai, Tar-Hwa Hsieh, Yen-Zen Wang, Ko-Shan Ho, Chia-Yun Chang

**Affiliations:** 1 Department of Chemical and Materials Engineering, National Kaohsiung University of Applied Sciences, 415, Chien-Kuo Road, Kaohsiung 80782, Taiwan; tsc0624@gmail.com (M.-J.T.); thh@cc.kuas.edu.tw (T.-H.H.); cyc@cc.kuas.edu.tw (C.-Y.C.); 2 Department of Chemical and Materials Engineering, National Yun-Lin University of Science and Technology, Yun-Lin 64002, Taiwan; wangzen@yuntech.edu.tw

**Keywords:** *N*-phenyl-*p*-phenylenediamine, aniline dimer, microwave, Pt-catalyst, fuel cell

## Abstract

The presence of *N*-phenyl-*p*-phenylenediamine (PPDA: a dimer of aniline) during microwave (MW) irradiation can significantly improve Pt-loading on the XC72 carbon matrix as a catalyst support of proton exchange membrane fuel cells (PEMFCs). PPDA is converted to an emeraldine base state during MW-assisted redox reaction, which is characterized by both FTIR and Raman spectra. The increased degree of conjugation from the formation of quinone-state of PPDA is confirmed by UV-VIS spectra. TEM micrographs and residue weights obtained from the TGA thermograms illustrate the particle size and Pt-loading percent of Pt nanoparticles (NPs) after MW irradiation, respectively. X-ray diffraction patterns indicate Pt NPs are successfully loaded on XC72 by MW irradiation corresponding to hydrothermal method. The single cell performance demonstrates an increasing power and maximum current density when Pt-catalyst of membrane exchanged assembly (MEA) is prepared by MW-assisted reduction in the presence of PPDA.

## 1. Introduction

In order to reduce the greenhouse gas emissions and smog pollution [1,2,3] generated from fossil fuels, hydrogen-based fuel cell technologies have been widely studied in various areas [4].

Recent studies focus on hydrogen (anode) and oxygen (cathode) based proton exchange membrane fuel cells (PEMFCs) under the consideration of its harmless water by-products and high power density with low noise and low operating temperatures. However, one of the main problems before commercializing PEMFCs is how to decrease the cost of Pt catalysts through catalytic efficiency and durability improvements under the harsh working conditions [5]. The performance of the PEMFCs also depends on the properties of the fuel gas flowing through the membrane electrode assembles (MEAs) [6] in which interfacial properties between the fuel gas, electrolyte, and catalyst, the so-called triple-phase boundary, play important roles on deciding the eventual power density.

Conducting carbon matrix materials are usually chosen as the catalyst-supporting materials for catalyst metals due to the excellent conductivity, high surface area, and low cost, which are all required for the commercialization of PEMFC. Some studies focused on the preparation of the conducting catalyst supports with nanoscale pores to trap and disperse the implanted Pt [7]. At present, catalyst material for MEA is prepared by loading nano-scale platinum (Pt) particles on the surface of conducting, nanostructured carbon black (Vulcan XC72). However, carbon support in the cathode can be subjected to severe corrosion in the presence of water, which can produce poisoning carbon monoxide and carbon dioxide easily at high performing temperatures [8,9,10], causing severe poisoning of the catalysts and shortening the lifetime of PEMFC. To obtain Pt-catalyst carbon-supporting materials with low corrosion at the working temperature has become an important topic in preparing PEMFC. 

Since Pt(IV) are easier to disperse than Pt particles, the alcohol solution of hydrogen hexachloroplatinate (IV) hexahydrate (H_2_PtCl_6_·6H_2_O) will be mixed with oligoaniline, which behaves as a microwave absorber [11,12,13,14,15], reductant, and captivator (through complexation) of Pt(IV) in the EG solution in the presence of conducting medium like XC72.

Qu et al. [16] prepared core-shell *N*-containing polyaniline/vulcan carbon composite structures by in situ polymerization, to improve the CO-poisoning resistance and catalytic efficiency of the Pt catalyst. Recently, carbonization on nanostructured nitrogen-containing catalyst support has created new perspectives in preparing nitrogen-containing conducting nanomaterial. Andre Wolz et al. [17] prepared a MEA made of polyaniline cathode and Nafion anode which achieved high power density with low Pt loading. Gavrilov et al. [18] prepared nitrogen-containing nanotube/nanosheet covered with carbonized polyaniline as a new carbonaceous support for Pt nanoparticles, which demonstrated significant ORR (The oxygen reduction reaction) in both acidic and alkaline media. Even though *N*-containing polyaniline can effectively absorb MW (microwave), the intractable and insoluble nature restricts its capability to carry out the redox reaction with Pt in the alcohol medium, and shortening the length of polyaniline to become oligoaniline does not lose its capability of absorbing MW, but can improve its solubility in the solvent medium that is capable to dissolve Pt(IV). 

*N*-phenyl-*p*-phenylenediamine (PPDA) is the simplest reduced state of oligoanilines that can complex with (capture) positive metal (Pt(IV)) ions for MW irradiation, and the followed redox reaction can be initiated by MW irradiation which can generate heat from the rotational friction of the polar amino-groups of PPDA, leading to the reduction of Pt(IV) similar to either hydrothermal (HT) or solvothermal treatment [19,20,21]. In addition, the presence of surface carbonyl groups of the XC72 can create H-bonds with PPDA, which is also an *N*-containing chemical that can extend the cycle life of the carbon-based XC72 support by its corrosion preventive (oxidation preventive) power. 

Furthermore, *N*-containing (PPDA H-bonded) XC72 support is able to capture Pt(IV) to disperse the reduced Pt particles more effectively through MW irradiation, saving the trouble of high temperature refluxing. *N*-containing carbonaceous supports have recently been reported to demonstrate significant ORR activity [22,23] which, combined with its special morphologies and properties, are directly related to the *N*-containing precursor it derived from [24,25].

Additionally, MW irradiation needs no input of solvent and can perform in a simple and easy manner, saving the troubles of solvent removal and sample drying. Most importantly, it can be carried out at room temperature with only several minutes of MW irradiation.

In this study, we try to prepare an *N*-containing XC72 as the conducting support for Pt NPs which can be deposited on its surface by facile MW irradiation in the presence of PPDA which behaves as a MW absorber, Pt(IV) captivator, and reductant. Various properties, like Pt loading percent of the catalyst support, electrochemical performance of the MEA, and the power density of the single cell, will be measured. 

## 2. Material and Methods

### 2.1. Materials

The *N*-phenyl-*p*-phenylenediamine (Tokyo Kasei Kogyo Co., Tokyo, Japan), hydrogen hexachloroplatinate (IV) hexahydrate (H_2_PtCl_6_·6H_2_O, Aldrich, St. Louis, MO, USA), ethylene glycol (EG, J.T. Baker^®^, Center Valley, PA, USA), and Vulcan XC72 (Cabot Corporation, Boston, MA, USA.) were used without further purification.

### 2.2. Preparation of Pt/XC72-PPDA-MW

Pt/XC72-PPDA-MW were prepared from 0.025 mmol of hexachloroplatinate (H_2_PtCl_6_) acid in 20 mL of ethylene glycol (EG), which contained 16 mg of suspended XC72 powders. The mixtures were under sonication for 15 min before 0.05 mmol (9.2 mg) of PPDA and a small amount of NaOH were introduced to maintain the pH of the EG solution above 11. The mixtures were subjected to MW irradiation. The obtained Pt/XC72 composites were isolated by filtration and washed with isopropanol, and dried in an oven at 60 °C overnight. The comparison experiment was carried out by the same procedures in the absence of PPDA. Another comparison experiment was performed by thermal treatment (refluxing) at 170 °C for 2 h without PPDA.

### 2.3. Basic Characterization

#### 2.3.1. Microwave Vector Network Analyzer, VNA

The absorbing effectiveness vs. frequency of PPDA was measured by a VNA (ZVB20) purchased from Rohde & Schwarz, München, Germany. 

#### 2.3.2. Microwave Oven

MW irradiation was carried out in a TMO-17MB model microwave oven with a fixed frequency of 2.45 GHz, provided by Tatung Co. (Taipei, Taiwan), with a maximum power of 700 W.

#### 2.3.3. FTIR Spectroscopy

The functional groups of PPDA and XC72 samples were characterized by FTIR spectroscopy. The FTIR spectra were recorded on an IFS3000 v/s Fourier-transform infrared spectrometer (Bruker Optics, Billerica, MA, USA) at room temperature, with resolution of 4 cm^−1^ and 16 scanning.

#### 2.3.4. Raman Spectroscopy

The Raman spectra of neat and microwaved PPDA samples were carried out by a Triax 550 spectroscope (Horiba, Kyoto, Japan) with a green laser light source at a wavelength of 520 nm. The samples were pressed into tablets before being subjected to the laser light source.

#### 2.3.5. UV-VIS-NIR Spectroscopy

The UV-VIS-NIR spectra of both PPDA and Pt/PPDA were obtained in ethylene glycol (EG) before and after MW irradiation from a Hitachi U-2001 spectrometer (Tokyo, Japan). A wavelength ranging from 300 to 1100 nm was used for each 5 mg sample dispersed in 100 mL of EG. 

#### 2.3.6. TEM (Transmission Electron Microscopy)

Samples taken pictures by field emission transmission electron microscope, HR-AEM (Hitachi FE-2000, Tokyo, Japan) were first dispersed in acetone and put on carbonic-coated copper grids dropwise before being subjected to the emission.

#### 2.3.7. TGA (Thermogravimetric Analysis)

The thermal degradation behavior of various Pt/XC72 series was recorded by TGA (TA SDT-2960, New Castle, DE, USA) thermograms. The amount of Pt deposited on the surface of the catalyst supports were characterized by the residual weights at 800 °C at 10 °C·min^−1^ under purging air.

#### 2.3.8. WXRD (Wide-Angle X-ray Diffraction: Powder X-ray Diffraction)

A copper target (Cu-Kα) Rigaku X-ray source with a wavelength of 1.5402 Å was used for X-ray diffraction. The scanning angle (2θ) started from 5° to 40°, with a voltage of 40 kV and a current of 30 mA, operated at 1°·min^−1^.

### 2.4. Electrochemical Characterization

The cyclic voltammetry method was used to determine the active electrochemical surface area of the catalyst supports in the electrode. The performance of the electrocatalyst support was tested with a three-electrode system. The square working electrode, with an area of 1.5 cm^2^, was prepared as follows: Ag/AgCl and platinum wire were used as the reference and counter electrodes, respectively. The electrochemical test was carried out in a potentiostat/galvanostat (Autolab-PGSTAT 30 Eco Chemie, Utrecht, The Netherlands) in 1 M H_2_SO_4_ solution and cyclic voltammograms (CV) were obtained with the scanning potential from −0.2 to 1.4 V at a sweeping rate of 50 mV·s^−1^. The catalyst ink was prepared by mixing 3 mg support powder in isopropanol and stirred until it became uniform. Subsequently, 5% Nafion solution was added into the mixture as binder and the mixture was ultrasonicated for 1 h, and the obtained ink was uniformly cast on the carbon paper for the CV test.

The electrochemical activities of the Pt/XC72-MW and Pt/XC72-PPDA-MW were measured using a rotating-disk electrode (RDE) operated at 1600 rpm in O_2_-saturated 1 M H_2_SO_4_. The oxygen reduction reaction (ORR) currents at the measured voltage range (0.5~1.0 V) for each electrocatalyst material were recorded.

### 2.5. MEA Preparation

A Nafion^®^ 212 sheet, purchased from Ion Power Inc. (New Castle, DE, USA), was used as the proton exchange membranes. In order to remove the surface organic impurities and to convert the membranes into protonated (H^+^) form, the Nafion-212 (4 × 4 cm^2^) membrane was treated at 70 °C in 5 wt % H_2_O_2_ aqueous solution for 1 h, followed by submerging in 1 M H_2_SO_4_ solution for 1 h and, subsequently, the treated membranes were dipped in distilled water for 15 min and stored in de-ionized water. The catalyst inks were prepared by mixing 20 mg of Pt/XC72-MW or Pt/XC72-PPDA-MW powders in isopropanol and mechanically stirred until it became uniform before 5% Nafion solution was added. Eventually, the catalyst mixture was ultrasonicated for 1 h followed by coating on both side of the treated Nafion sheet in a dropwise fashion as anode and cathode electrodes (2 × 2 cm^2^), respectively, and hot-pressed at 140 °C with a pressure force of 70 kg·cm^−2^ for 5 min to obtain the MEA.

### 2.6. Single-Cell Performance Testing

The MEA was installed in a fuel cell test station for testing using the single-cell test equipment (model FCED-P50; Asia Pacific Fuel Cell Technologies, Ltd., Miaoli, Taiwan). The active cell area was 2 cm × 2 cm. The temperatures of anode, cell, and cathode and humidifying gas were all maintained at around 70 °C. The flow rates of the anode input H_2_ and the cathode input O_2_ fuel were set at 100 and 200 mL·min^−1^, respectively. To test the electrochemical performance of the Pt/XC72-MW and Pt/XC72-PPDA-MW catalyst in the individual MEAs, both polarization curves (I–V) and output powers were measured.

## 3. Results and Discussion

### 3.1. Microwave Absorption of PPDA

Usually, the oxidation of the amino group of aniline can only be performed in strongly acidic conditions by a strong oxidant, like persulfates or peroxides, and the aniline monomers can be polymerized (oxidized) into polyanilines [26,27,28]. Theoretically, the strong base condition created by the addition of aqueous sodium oxide (pH = 11) in the PtCl_6_^−2^ aqueous solution is unfavorable for the oxidation of PPDA, which is actually an aniline dimer. Therefore, the redox reaction must be carried out at high temperature (170 °C) fluxing (solvothermal or HT method) for 2 h in the reductant solvents, like alcohol or ethylene glycol (EG), which is often seen in the conventional preparation of Pt nano-particles (NPs).

The heat generated by MW absorption of the amino-containing compounds is another facile way to create a high-temperature environment for the redox reaction, which leads to the formation of Pt NPs in a very short time.

**Figure 1:**
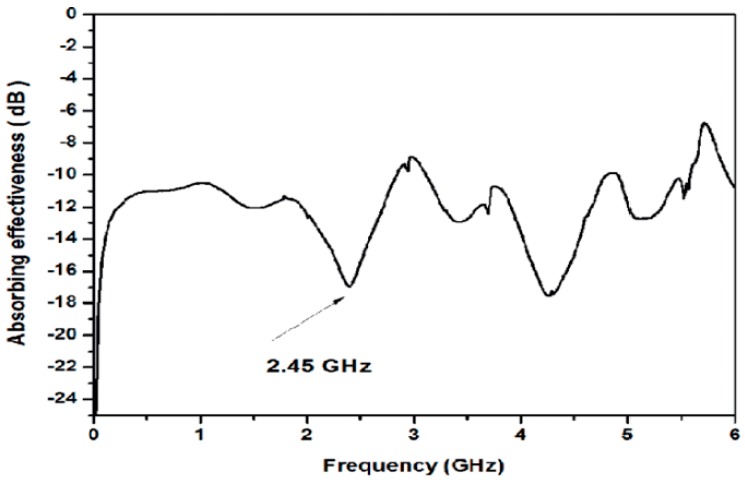
Microwave spectrum of neat PPDA.

PPDA, which is an aniline dimer, is thought to be an effective microwave absorber than alcohol-type chemicals, like EG, which does not absorb microwaves and generates no heat during MW irradiation. Measured by a surface thermometer, the surface temperature of each sample was maintained below 80 °C during MW processing. The preliminary experiment demonstrating the microwave absorbing capability of PPDA vs. frequency in Figure 1, which illustrates several characteristic absorbing frequencies of PPDA, especially the frequency around 2.45 GHz. At this frequency, an effectiveness of 17 dB was found, meaning more than 90% of MW was absorbed by PPDA and 2.45 GHz matches the frequency provided by the common microwave oven. In other words, the reducing power of PPDA can be activated by the microwave absorption, which can initiate and finish the redox reaction between PPDA and Pt(IV) in 90 s. All of the following Pt catalysts were prepared in a regular microwave oven with tunable power (700 W, maximum) but with a fixed frequency of 2.45 GHz.

**Scheme 1:**
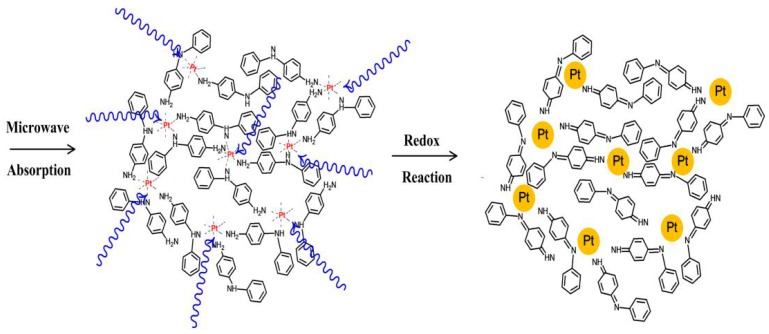
Schematic diagram of the microwave-assisted redox reaction between PPDA and XC72.

Another benefit of using PPDA as the reductant is its capability of forming H bonds with XC72 at one end (which will be confirmed by IR-spectrum) and coordinating with Pt(IV) at the other, inducing a fast coordination-oxidation nucleation process [29] upon MW irradiation. In the beginning, the PPDA was mixed in the stirring H_2_PtCl_6_/NaOH aqueous solution (pH = 11) and immediately a dark colored solution was formed due to the strong coordination between PPDA and Pt(IV). The PPDA in the complexes played the role as the reductant and the Pt(IV) was reduced to Pt NPs in 90 s. With MW irradiation, the complex converted completely into Pt NPs and PPDA was converted into an EB (emeraldine base) state, as depicted in Scheme 1. The Pt quickly accumulated into spherical NPs and combined tightly on the PPDA-XC72 matrix, which was obtained by the H bonding between PPDA (–NH–) and XC72 (–C=O). 

### 3.2. FTIR Spectroscopy

**Figure 2:**
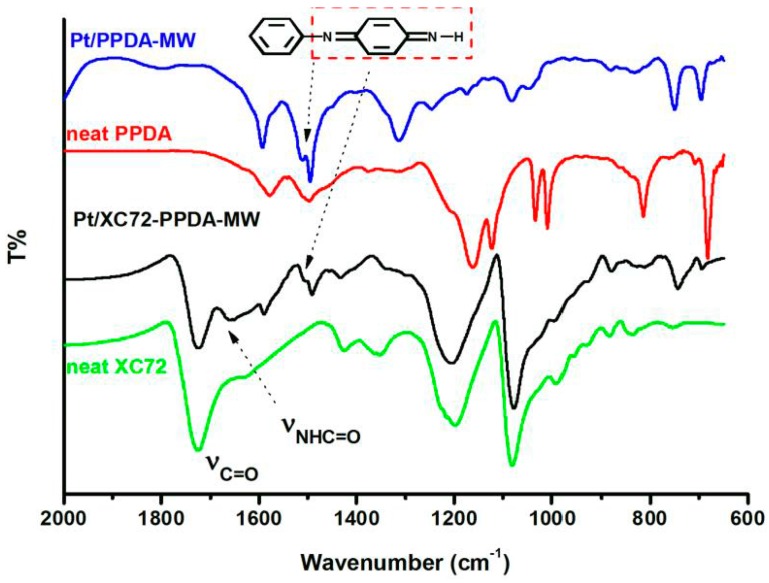
FTIR spectra of neat PPDA and Pt/XC72-PPDA-MW.

**Scheme 2:**
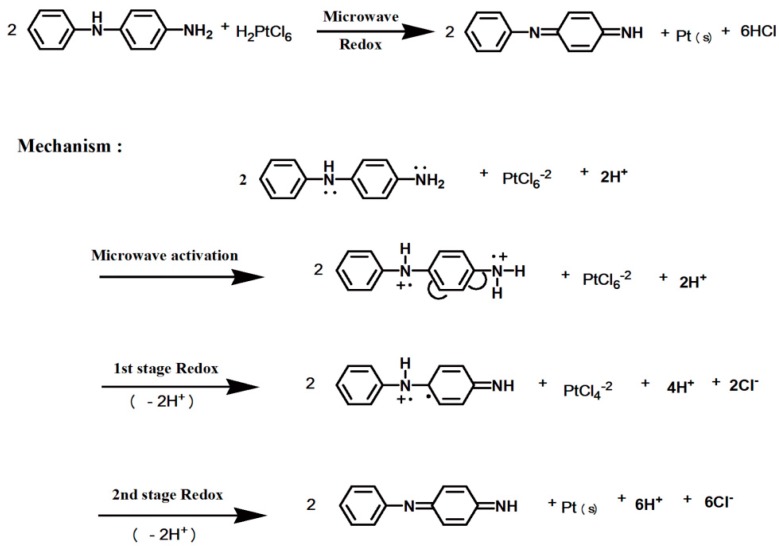
Mechanism of the microwave assisted redox reaction between PPDA and XC72.

The FTIR spectra of neat PPDA, XC72, and coordinated with Pt(IV), are shown in Figure 2, which can not only be used to explain the redox reaction between PPDA and Pt(IV), but can illustrate the possible side reaction occurring to XC72 under the irradiation of MW. Scheme 2 was constructed according to the results obtained from the IR spectra in Figure 2. The detailed explanation follows.

The IR spectrum of neat PPDA in Figure 2 demonstrated peaks at 1576 and 1492 cm^−1^, corresponding to the benzenoid ring stretching of the secondary and primary amines of neat PPDA, respectively. After performing the redox reaction with Pt(IV) by MW irradiation, an additional peak at 1515 cm^−1^ in Figure 2 appears, which reveals that part of the benzene rings connecting to the primary amines were oxidized and quinonized via the redox reaction with Pt(IV) after microwaving. A redox mechanism was postulated and demonstrated in Scheme 2, which explains the oxidative paths of both secondary and primary amines under MW irradiation during which the Pt(IV) are able to extract electrons from PPDA and reduce into NPs spontaneously. The presence of a strong base condition of NaOH_(aq)_ (pH = 11) can immediately remove the proton byproducts by neutralization to avoid the occurrence of a reverse reaction and effectively increases the yield of Pt NPs. This tells why, even in the HT or solvothermal treatment of reducing Pt(IV) in the presence of alcohol or EG, we still need to keep the pH value of the aqueous Pt(IV) solution as high as 11, since any redox reaction between Pt(IV) and active H-containing organic compounds will generate protons which can be removed very quickly by neutralization with a strong base to keep the redox reaction going in the forward direction. The H- of the amine group is more likely to be removed, compared to that of alcohol or EG. Therefore, PPDA in the mixture not only plays the role of a MW absorber, but also the role of effective reductant, which can convert its H- into a proton upon absorbing MWs.

The IR-spectra of Pt/PPDA-MW and Pt/XC72-PPDA-MW were also illustrated in Figure 2 in which we still found a peak at 1515 cm^−1^ for both spectra after MW irradiation. When we compare this to the spectrum of neat XC72, the carbonyl peak (1727 cm^−1^) of neat XC72 is split into two peaks after MW irradiation, an additional, broad band at around 1660 cm^−1^ is created, assigned to the amide groups derived from the reaction between the amine of PPDA and carbonyl of XC72 during MW irradiation. The formation of amide covalent bond and the H bondings between PPDA(EB) and XC72 strongly indicate that we do obtain an *N*-containing conducting PPDA-XC72 matrix by facile MW treatment.

### 3.3. Raman Spectroscopy

**Figure 3:**
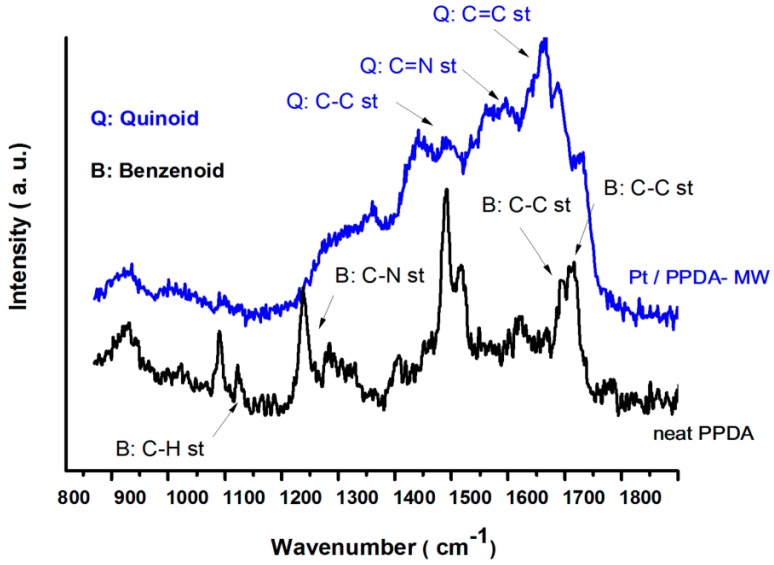
Raman spectra of neat PPDA and Pt/XC72-PPDA-MW.

Since both benzene and quinone groups are partly made of carbons, they can be characterized by Raman spectroscopy as well. Furthermore, Raman spectra were used to analyze the molecularly structural variation of PPDA upon MW irradiation. We checked the Raman spectra of both neat PPDA and Pt/PPDA-MW to characterize the degree of quinonization of PPDA (EB state) during the redox reaction assisted by MW and demonstrated in Figure 3. The Raman spectrum of neat PPDA shows the corresponding peaks of benzenoid before microwaving in the presence of Pt(IV), whereas a large number of quinoid-related peaks generated after MW irradiation, according to Figure 3, include the stretching modes of –C=C– and –C=N–, which constitute the major part of quinone groups of PPDA(EB). The experiments were performed in the absence of XC72, avoiding the strong absorption of its abounding unsaturated, saturated carbons. The Raman spectrum, which is another type of vibrational spectroscopy, leads to the same conclusions obtained from IR spectroscopy.

### 3.4. UV-VIS Spectroscopy

**Figure 4:**
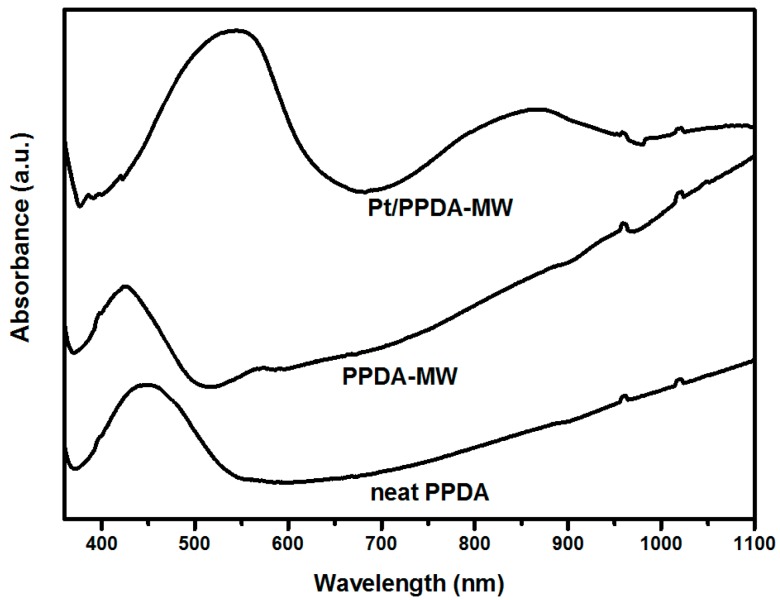
UV-VIS spectra of neat PPDA, PPDA-MW, and Pt/PPDA-MW.

The two phenyl rings of PPDA are straightly conjugated with each other due to a disruption of the bridge secondary amine and the λ_max_ is about 450 nm, corresponding to the π to π* transition of neat PPDA, as seen in Figure 4. The variation of the conjugation length of PPDA before and after MW irradiation can be well-monitored by the UV-VIS-NIR spectroscopy. The red- or blue-shifting of the λ_max_ is directly related to the lengthening or shortening of conjugation length of PPDA. From Figure 4, we understand the MW irradiation carried out in the absence of Pt(IV) caused the damage of the conjugation and demonstrated a blue-shift of λ_max_ to 427 nm from 450 nm, indicating the MW can be absorbed by PPDA, which eventually shortened the conjugation chain length. In other words, if the excited PPDA (MW absorbed) is not going conducting the redox reaction with Pt(IV), side reactions might occur, leading to the decrease of conjugation length. The MW is just like the catalyst that catalyzes the redox reaction between PPDA and Pt(IV). Consequently, PPDA can be oxidized by Pt(IV) and become PPDA(EB) under the catalysis of MW irradiation. Additionally, the –C=N– double bond of the resultant quinonized PPDA(EB) became the conjugation bridge of the phenyl and quinone rings and significantly improved the conjugation length, resulting in the far and broad red-shift λ_max_ of 550 nm according to Figure 4. The presence of Pt-nanoparticles can also be confirmed by the plasmonic absorption peak centered at 870 nm of Pt/PPDA-MW.

### 3.5. Transmission Electronic Microscopy (TEM)

In most of the cases, the catalytic efficiency of the implanted Pt particles on either hydrogen oxidation or oxygen reduction in a fuel cell can be significantly reduced if they are aggregated. The Pt loading needs to be carried out in a way that can prevent the accumulation of large Pt NPs in order to provide as much active surface area as possible for the redox reaction.

**Figure 5:**
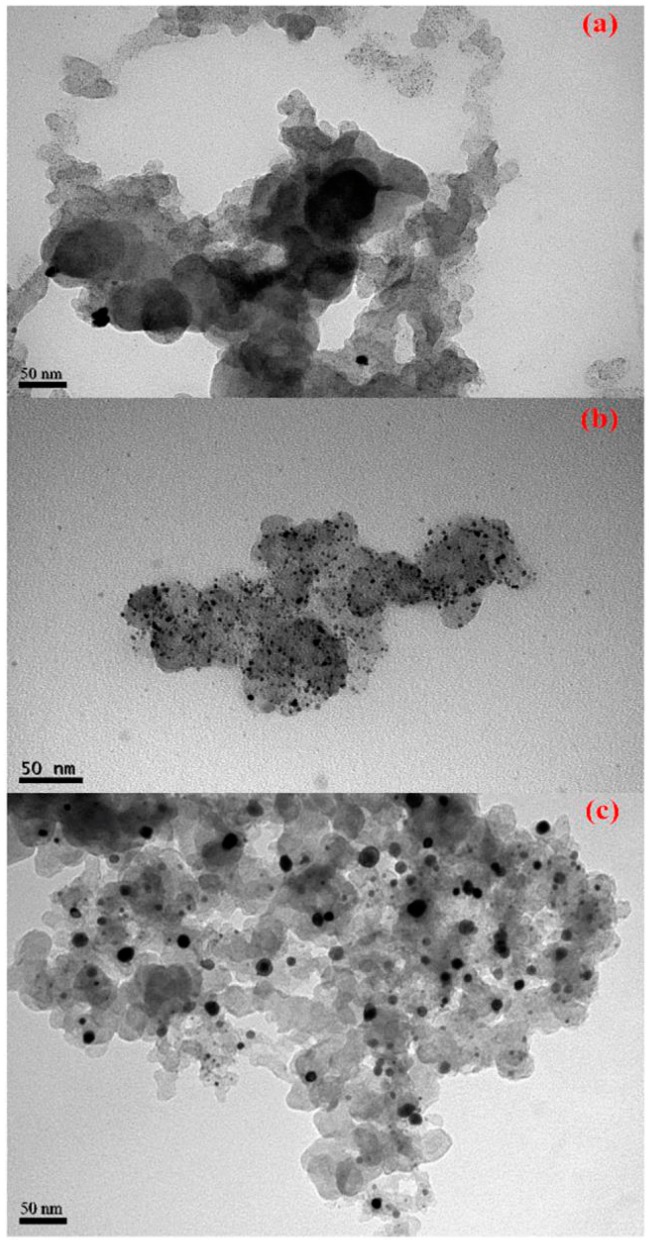
TEM micrographs of various Pt-loaded PANFs (**a**) Pt/XC72-HT; (**b**) Pt/XC72-MW; and (**c**) Pt/XC72-PPDA-MW.

TEM micrographs demonstrated in Figure 5 are used to check the particle size and the distribution of implanted Pt NPs. Obviously, all Pt NPs were well-distributed in the XC72 matrix; they were either prepared with HT or MW methods. However, the particle size of the implanted Pt NPs turned out to be quite different. The experiment with Pt(IV) refluxing in the EG solvent (HT treatment) in the presence of XC72 provided tiny particles with a size around 1–2 nm of implanted Pt NPs in Figure 5a. To check the effect of MW irradiation, we prepared the same mixture of Pt(IV), XC72, and EG solvent in the absence of PPDA, and treated with MW irradiation. Its TEM micrograph in Figure 5b demonstrates larger Pt particles with sizes of around 5–7 nm in the XC72 matrix. It seems that MW improves the redox reaction between Pt(IV) and EG like solvothermal heating (Figure 5a). Eventually, PPDA, which owns high, strong MW absorbing characteristics, as illustrated in Figure 1 was introduced to the same system. The TEM micrograph of Pt/XC72-PPDA-MW demonstrated in Figure 5c reveals a higher concentration of Pt and the largest particle size of around 10 nm, compared to that of Figure 5a,b, indicating the enhancement of Pt production in the presence of MW-absorbable PPDA. The larger size of reduced Pt in the presence of PPDA possibly comes from the easy accumulation of PPDA molecules, which are prone to associate through the formation of intermolecular H bondings when solvent is evaporated during MW irradiation.

The exact weight of the Pt loading percent will be measured by TGA thermograms in the following discussion.

### 3.6. Thermogravimetric Analysis (TGA)

**Figure 6:**
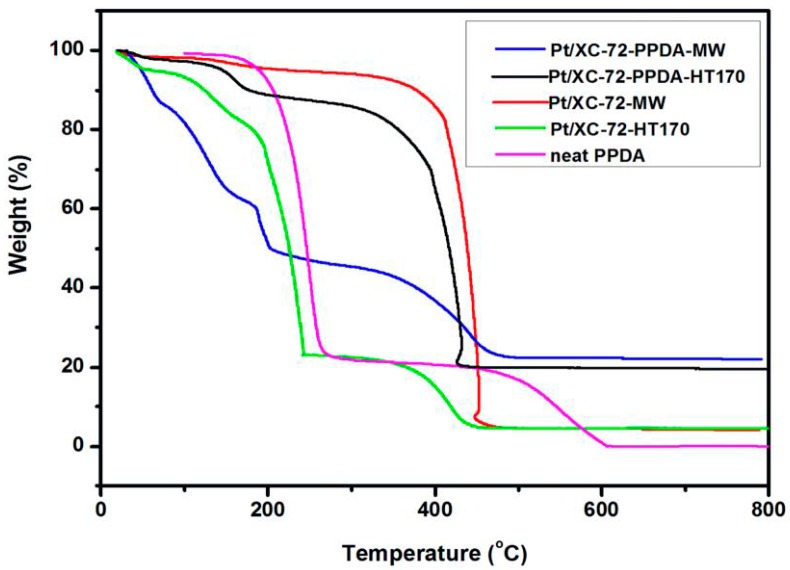
TGA thermograms of neat PPDA and various Pt-loaded supports.

Another factor that can improve the efficiency of the Pt catalyst is the weight besides the factor of active surface area. It can be measured by burning out all of the non-metal materials at high temperature, which can be carried out in a thermogravimetric analysis. The residue weight of the TGA thermogram was used to represent the exact Pt loading percent of various types of Pt catalyst. The blank testing was performed for the neat PPDA, which decomposed entirely after 600 °C in purging air, referring to Figure 6. Reasonably, the residual weight percent after 750 °C is defined as the Pt loading percent on the XC72.

The TGA thermograms demonstrated in Figure 6 clearly illustrate that PPDA is the key material to obtain high Pt-loading percent. In the absence of PPDA, both microwave-assisted and HT treatments can only produce 4.11% of Pt even though EG was considered to be the reductant for Pt(IV) during microwave irradiation or HT treatment, as shown in Figure 6.

The Pt-loading percent was greatly improved when PPDA was introduced into the reduction system by either HT heating or MW irradiation. The Pt-loading percent was increased to 19.47% when PPDA was present during HT reduction at 170 °C. More effectively, the addition of PPDA in the microwave-assisted reduction tremendously improved the Pt-loading percent to 21.98% from 4.11% when only EG was the only reductant medium. Indeed, the high Pt reduction percent does not guarantee the high power density and current of the final single cell. We need the Pt catalyst to be well-dispersed to increase its activity area, which cannot be monitored by TGA thermograms, but TEM micrographs, which was already discussed in the previous section.

### 3.7. X-ray Diffraction Pattern of the Electrocatalyst Electrode Materials

The average implanted Pt crystal size for each electrocatalyst electrode material is calculated by the Debye-Scherrer equation based on the X-ray diffraction (220) plane of crystalline Pt prepared by HT and MW treatment, respectively:

$$d=\frac{k\lambda }{\beta \cos\theta }$$

where *k* is a coefficient (0.9), λ is the wavelength of the X-rays (0.1541 nm for CuKα), β is the full-width half-maximum (FWHM) of the respective diffraction peaks measured at 2θ (in radians), and θ is the diffraction angle of the peak in degrees. The calculated mean Pt crystallite sizes obtained from XRD patterns for each electrocatalyst are listed in Table 1 for HT and MW-loaded Pt, respectively. The particle sizes obtained are smaller than that from TEM micrographs since only the coherent domain can be detected by X-ray diffraction. From the calculated particle sizes and Pt loading percent obtained from TGA thermograms, we can estimate the surface area of the implanted Pt atoms per unit catalyst support those listed in fourth column of Table 1. We can calculate the overall surface area by dividing the data presented in this column with the Pt-loading percentage from the residue weight percent from TGA thermograms. The overall surface area of the Pt loaded by the HT method is 193.23/19.47 = 9.92 and by MW irradiation is 342.6/21.98 = 15.58, which indicates the MW method demonstrates 1.57 times higher surface area of loaded Pt elements than that of HT.

### 3.8. Electrochemical Analysis

#### 3.8.1. Cyclic Voltammetry (CV)

**Figure 7:**
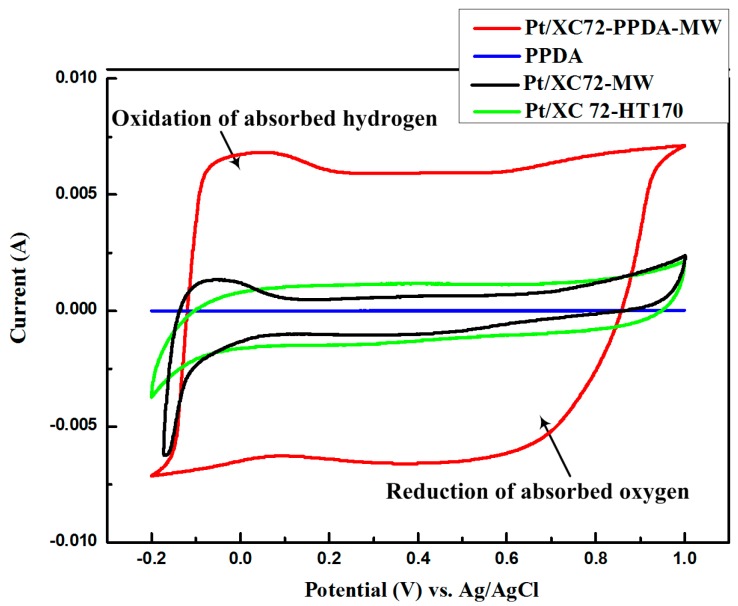
The current-voltammogram curves of PPDA, Pt/XC72-MW, Pt/XC72-HT170, and Pt/XC72-PPDA-MW.

The peak at around 0.60 V shown in Figure 7 for Pt/XC72-PPDA-MW is considered to be the redox behavior [30] and no significant peak can be seen for the CV curve of neat XC72. The PPDA can be also converted into quinone-like structures (EB state) during the redox reaction with Pt(IV), as already confirmed by the FTIR and Raman spectra, which can create more extended π-to-π* conjugation with the benzene ring of PPDA [31,32] and effectively increases the numbers of active oxidation sites to radical PPDA on the electrode surface. It is also considered as the rate-determining step in potentiodynamic loading of Pt atoms. The presence of the oxidation peak for PPDA indirectly illustrates some of the N atoms are still preserved in the microwaved PPDA, which can improve the capturing ability on either H^+^ or Pt(IV) for better proton conductivity and a higher degree of Pt-loading in the electrodes [33].

Additionally, the symmetric curve of PPDA shows that the catalyst support will experience a stable redox reaction at a high number of cycles, revealing that more active and stable redox reactions can occur when they are loaded with Pt and fabricated into electrodes of MEAs.

Only the specific active surface area of Pt/XC72-PPDA-MW is calculated to be 2.21 m^2^/g. Combined with the 21.98% obtained from TGA thermogram in Figure 6, the total active area of Pt/XC72-PPDA-MW is about 48.58 m^2^ per 100 g sample.

#### 3.8.2. Oxygen Reduction Reaction (ORR) Performance

**Figure 8:**
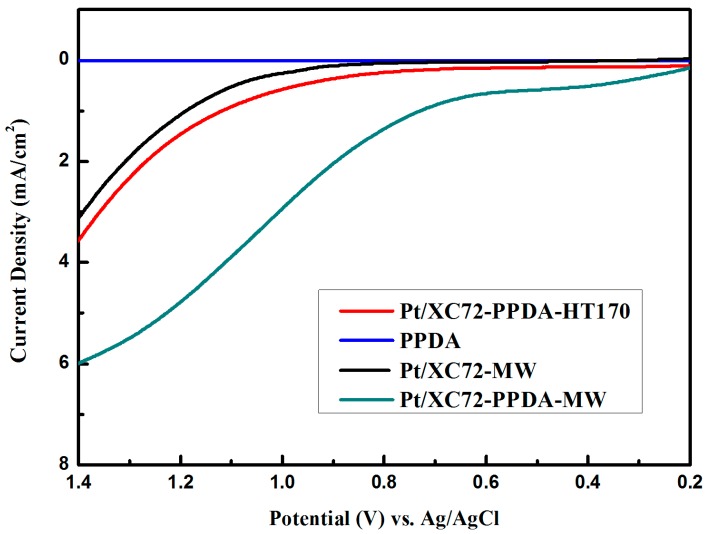
The ORR curves of neat PPDA, Pt/XC72-MW, Pt/XC72-HT and Pt/XC72-PPDA-MW.

To evaluate the electrocatalytic activity of different Pt electrocatalysts based on XC72 support, their ORRs are measured and shown in Figure 8. Since there is no redox activity for neat PPDA, the reduced current almost vanishes. The reduced current of both Pt/XC72-MW and Pt/XC72-HT systems were smaller than that of the microwaved Pt/XC72-PPDA-MW system, according to Figure 8, indicating better ORR capability for the Pt/XC-PPDA-MW catalyst and the necessity of the presence of PPDA during preparation of the Pt catalyst under MW irradiation. It is understood some PPDA was converted to the EB state, which can create H bonding with carbonyl containing XC72, and others formed amide bonding with it after MW irradiation. Both contribute *N* atoms to the Pt electrode and demonstrated better ORR power.

#### 3.8.3. Single Cell Performance Analysis

**Figure 9:**
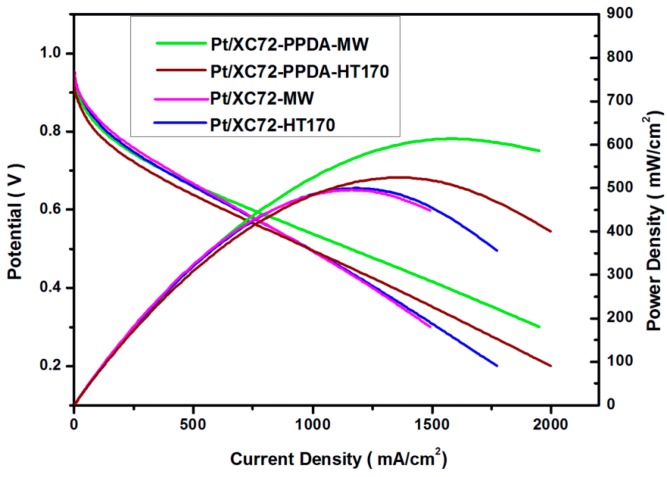
Electrocatalytic polarization and power density curve of PEMFC based on different catalyst supports. The fuel cell temperature was at 70 °C. The flow rates of H_2_ and O_2_ flows are set at 0.1 and 0.2 L·min^−1^, respectively.

MEAs based on different methods of preparation of the Pt catalyst are assembled into single cells and their electrochemical performances are evaluated by measuring their current density, voltage, and power density in Figure 9. The presence or absence of the PPDA during microwave reduction of Pt plays a key role of the eventual power density of the single cell. When there was no PPDA mixing with EG in either microwave-assisted reduction or HT treatment, the assembled single cell demonstrated similar maximum power density (*P*_max_) below 500 mW·cm^−2^ and similar maximum current (*I*_max_) at about 1300 mA·cm^−2^. When a slight amount of PPDA was incorporated in the preparation of Pt-catalyst by HT treatment, the *P*_max_ is increased to be over 500 mW·cm^−2^. However, when Pt-catalyst was prepared under microwave irradiation in the presence of PPDA, the *P*_max_ jumped over 600 to 620 mW·cm^−2^ and the *I*_max_ was extended to 1520 mA·cm^−2^, according to Figure 9. The presence of PPDA in both microwave-assisted reduction and HT treatment can effectively improve the Pt loading percent and decrease the effect of the concentration polarization according to Figure 9. Additionally, the cell experiences only slight voltage decaying with increasing current density due to little concentration polarization that could be attributed to the *N*-containing PPDA. Both higher *P*_max_ and *I*_max_ for the presence of additional PPDA shown in Figure 9 indicate that, to obtain large amounts of Pt catalyst in an easy and quick way, the reduction with microwave irradiation in the presence of an effective microwave-absorbable reductant like PPDA ought to be carried out. The microwave method also saves the trouble of separating Pt from water or solvent and can be applied directly to manufacture the MEA.

## 4. Conclusions

Based on avoiding the tedious and low yield HT or solvothermal preparation of Pt loading, we prepared Pt-loading XC72 by MW irradiation in the presence of PPDA which is also an *N*-containing chemical that can effectively improve the catalytic activity of Pt. 

PPDA was found to be able to convert to quinonized state under MW irradiation and improve its redox reaction with Pt(IV), characterized by FTIR and Raman spectra, respectively. The formation of quinone can extend the conjugation path with the benzene ring of PPDA and effectively increase the conjugation chain length as confirmed by UV-VIS-NIR spectra. TEM micrographs show well-dispersed and tiny Pt particles on the XC72 and residue weight of TGA thermograms illustrated higher Pt-loading weight after MW irradiation in the presence of PPDA. X-ray diffraction patterns indicate same type of Pt atoms similar to hydrothermal treatment were loaded on XC72 and the particles size and active surface area are calculated as well. The CV diagram reveals the presence of the quinone structure of PPDA during the redox reaction. The single cell performance demonstrates an increasing power and maximum current density when the Pt catalyst was prepared by facile MW irradiation in the presence of PPDA.

The presence of conjugated amino-structure is found to be able to absorb MW and assist the sequential redox reaction with active metal ions. The facile MW approach can provide another choice in the Pt loading on the conducting support, like XC72, for the preparation of the Pt catalyst electrode of the MEA. In the future, we will try other amino-containing aromatic compounds, like aniline or phenylenediamine, as the MW absorber and active reductant for Pt reducing to find the importance of conjugation length and amino-concentration on the MW absorbance and Pt loading.

## Figures and Tables

**Table 1:** Properties of Pt loaded on XC72 with different methods.

Method	Pt particle size ^a^ (nm)	Pt residual weight ^b^ %	Pt surface area per unit support ^c^ (cm^2^·mg^−1^)
HT	3.5	19.47	193.23
MW	5.2	21.98	342.62

^a^ Obtained from X-ray diffraction; ^b^ The Pt percent is obtained from the residual weight percent of the TGA thermogram; ^c^: To estimate the surface area of Pt per unit catalyst support, we can assume every Pt particle is separated from each other and is spherical with a diameter (particle size) listed in the second column. The residual percent of loaded Pt listed in the third column is considered as the weight (W) and the density (ρ) of loaded Pt is about 21.45 g·cm^−3^. The active surface area index can be estimated as the summation of surface area of each particle: $\left(W/(100-W)/\rho /\left(\left(4\pi /3\right)R^{3}\right)\right)*4\pi R^{2}=3W/\left(100-W\right)/21.45/R$, where $\left(\left(4\pi /3\right)R^{3}\right)$ and $4\pi R^{2}$ are volume and surface area of single particle, respectively.

## References

[1] Brentner L.B., Peccia J., Zimmerman J.B. (2010). Challenges in Developing Biohydrogen as a Sustainable Energy Source. Environ. Sci. Technol..

[2] Das D. (2009). Advances in bio-hydrogen production processes: An approach towards commercialization. Int. J. Hydrog. Energy.

[3] Logan B.E. (2004). Biologically extracting energy from wastewater: Biohydrogen production and microbial fuel cells. Environ. Sci. Technol..

[4] Ho L.Q., Sugano Y., Yoshikawa H., Saito M., Tamiya E. (2011). Structural assembly effects of Pt nanoparticle-carbon nanotube-polyaniline nanocomposites on the enhancement of biohydrogen fuel cell performance. Electrochim. Acta.

[5] He D., Zeng C., Xu C., Cheng N., Li H., Mu S., Pan M. (2011). Polyaniline-functionalized carbon nanotube supported platinum catalysts. Langmuir.

[6] Cindrella L., Kannan A.M. (2009). Membrane electrode assembly with doped polyaniline interlayer for proton exchange membrane fuel cells under low relative humidity conditions. J. Power Sources.

[7] Michel M., Ettingshausen F., Scheiba F., Wolz A., Roth C. (2008). Using layer-by-layer assembly of polyaniline fibers in the fast preparation of high performance fuel cell nanostructured membrane electrodes. Phys. Chem. Chem. Phys..

[8] Kinoshita K. (1988). Carbon: Electrochemical and Physicochemical Properties.

[9] Kinoshita K., Bett J.A.S. (1973). Potentiodynamic analysis of surface oxides on carbon blacks. Carbon.

[10] Pyun S.I., Lee E.J., Kim T.Y., Lee S.J., Ryu Y.G., Kim C.S. (1994). Role of surface oxides in corrosion of carbon black in phosphoric acid solution at elevated temperature. Carbon.

[11] Chandrasekhar P., Naishadham K. (1999). Broadband microwave adsorption and shielding properties of a poly(aniline). Synth. Met..

[12] Abbas S.M., Chandra M., Verma A., Chatterjee R., Goel T.C. (2006). Complex permittivity and microwave adsorption properties of a composite dielectric absorber. Compos. Part A Appl. Sci. Manuf..

[13] Faez R., Martin I.M., De Paoli M.-A., Rezende M.C. (2001). Microwave properties of EPDM/PAni-DBSA blends. Synth. Met..

[14] John H.R., Thomas M., Jacob J., Mathew K.T., Joseph R. (2007). Conducting polyaniline composites as microwave absorbers. Polym. Comp..

[15] Oyharcabal M., Olinga T., Foulc M.-P., Lacomme S., Gontier E., Vigneras V. (2013). Influence of the morphology of polyaniline on the microwave absorption properties of epoxy polyaniline composites. Comp. Sci. Technol..

[16] Qu B., Xu Y., Deng Y., Peng X., Chen J., Dai L. (2010). Polyaniline/carbon black composite as Pt electrocatalyst supports for methanol oxidation: Synthesis and characterization. J. Appl. Polym. Sci..

[17] Mentus S., Ćirić-Marjanović G., Trchová M., Stejskal J. (2009). Conducting carbonized polyaniline nanotubes. Nanotechnology.

[18] Gavrilov N., Dašić-Tomić M., Pašti I., Ćirić-Marjanović G.S. (2011). Carbonized polyaniline nanotubes/nanosheets-supported Pt nanoparticles: Synthesis, characterization and electrocatalysis. Mater. Lett..

[19] Yan X., Liu H., Liew K.Y. (2001). Size control of polymer-stabilized ruthenium nanoparticles by polyol reduction. J. Mater. Chem..

[20] Liu Z., Gan L.M., Hong L., Chen W., Lee J.Y. (2005). Carbon-supported Pt nanoparticles as catalysts for proton exchange membrane fuel cells. J. Power Sources.

[21] Esmaeilifar A., Rowshanzamir S., Eikani M.H., Ghazanfari E. (2010). Synthesis methods of low-Pt-loading electrocatalysts for proton exchange membrane fuel cell systems. Energy.

[22] Higgins D.C., Meza D., Chen Z. (2010). Nitrogen-doped carbon nanotubes as platinum catalyst supports for oxygen reduction reaction in proton exchange membrane fuel cells. J. Phys. Chem. C.

[23] Gong K., Du F., Xia Z., Durstock M., Dai L. (2009). Nitrogen-doped carbon nanotube arrays with high electrocatalytic activity for oxygen reduction. Science.

[24] Chen Z., Higgins D., Chen Z. (2010). Electrocatalystic activity of nitrogen doped carbon nanotubes with different morphologies for oxygen reduction reaction. Electrochim. Acta.

[25] Chen Z., Higgins D., Tao H., Hsu R.S., Chen Z.J. (2009). Highly active nitrogen-doped carbon nanotubes for oxygen reduction reaction reaction in fuel cell applications. J. Phys. Chem. C.

[26] Wang Y.Z., Tsai M.J., Hsieh T.H., Tseng P.H., Ho K.S. (2015). Studies on the 1D polyanilines prepared with *N*-dodecylbenzenesulfonic and camphorsulfonic acids. Polym. Int..

[27] Wu R.H., Tsai M.J., Ho K.S., Wei T.E., Hsieh T.H., Han Y.K., Kuo C.W., Tseng P.H., Wang Y.Z. (2014). Sulfonated Polyaniline Nanofiber as Pt-Catalyst Conducting Support for Proton Exchange Membrane Fuel Cell. Polymer.

[28] Wang Y.Z., Chang K.J., Hung L.F., Ho K.S., Chen J.P., Hsieh T.H., Chao L. (2014). Carboxylated Carbonized Polyaniline nanofibers as Pt-Catalyst Conducting Support for Proton Exchange Membrane Fuel Cell. Synth. Met..

[29] Yang S.W., Hu C.S., Liu D., Zhang T.T., Guo T.T., Liao F. (2014). Synthesis of Platinum Nanoparticles-Decorated Poly(*p*-Phenylenediamine) Colloids with a High Performance for Methanol Electrocatalysis for Direct Methanol Fuel Cells. J. Clust. Sci..

[30] Genies E.M., Boyle A., Lapkowski M., Tsintavis C. (1990). Polyaniline: A historical survey. Synth. Met..

[31] Zhao M., Wu X., Cai C. (2009). Crystal chemistry and dehydrogenation/rehydrogenation properties of perovskite hydrides RbMgH_3_ and RbCaH_3_. J. Phys. Chem. C.

[32] Hsu C.H., Liao H.Y., Kuo P.L. (2010). Aniline as a Dispersant and Stabilizer for the Preparation of Pt Nanoparticles Deposited on Carbon Nanotubes. J. Phys. Chem. C.

[33] Maiyalagan T., Viswanathan B., Varadaraju U.V. (2005). Nitrogen containing carbon nanotubes as supports for Pt—Alternate anodes for fuel cell applications. Electrochem. Commun..

